# Human resource management in Ethiopian public hospitals

**DOI:** 10.1186/s12913-022-08046-7

**Published:** 2022-06-10

**Authors:** Philipos Petros Gile, Joris van de Klundert, Martina Buljac-Samardzic

**Affiliations:** 1 Erasmus School of Health Policy and Management, Erasmus University Rotterdam, The Netherlands and Higher Education Institutions’ Partnership, CMC Road, PO Box 14051, Addis Ababa, Ethiopia; 2 Prince Mohammad Bin Salman College (MBSC) of Business and Entrepreneurship, 7082-BayLaSun-Juman St. Unit No. 1, King Abdullah Economic City, 23964-2522 Saudi Arabia, Kingdom of Saudi Arabia; 3 Erasmus University Rotterdam, Erasmus School of Health Policy and Management, PO Box 1738, 3000, DR Rotterdam, The Netherlands

**Keywords:** Ethiopian public hospitals, Strategic human resource management, Hospital reform, Autonomy, Contextual factors

## Abstract

**Background:**

In Ethiopia, public hospitals deal with a persistent human resource crisis, even by Sub-Saharan Africa (SSA) standards. Policy and hospital reforms, however, have thus far resulted in limited progress towards addressing the strategic human resource management (SHRM) challenges Ethiopia’s public hospitals face.

**Methods:**

To explore the contextual factors influencing these SHRM challenges of Ethiopian public hospitals, we conducted a qualitative study based on the Contextual SHRM framework of Paauwe. A total of 19 structured interviews were conducted with Chief Executive Officers (CEOs) and HR managers from a purposive sample of 15 hospitals across Ethiopia. An additional four focus groups were held with professionals and managers.

**Results:**

The study found that hospitals compete on the supply side for scarce resources, including skilled professionals. There was little reporting on demand-side competition for health services provided, service quality, and service innovation. Governmental regulations were the main institutional mechanism in place. These regulations also emphasized human resources and were perceived to tightly regulate employee numbers, salaries, and employment arrangements at detailed levels. These regulations were perceived to restrict the autonomy of hospitals regarding SHRM. Regulation-induced differences in allowances and external employment arrangements were among the concerns that decreased motivation and job satisfaction and caused employees to leave. The mismatch between regulation and workforce demands posed challenges for leadership and caused leaders to be perceived as incompetent and unable when they could not successfully address workforce needs.

**Conclusions:**

Bottom-up involvement in SHRM may help resolve the aforementioned persistent problems. The Ethiopian government might better loosen regulations and provide more autonomy to hospitals to develop SHRM and implement mechanisms that emphasize the quality of the health services demanded rather than the quantity of human resources supplied.

**Supplementary Information:**

The online version contains supplementary material available at 10.1186/s12913-022-08046-7.

## Introduction

The genesis of the human resources for health crisis in Sub-Saharan Africa (SSA) is complex and context specific, even if common factors are applicable across Africa [1–3]. SSA carries 24% of the global burden of disease, but only 3.5% of the global health workforce works in this region [2, 4, 5]. Ethiopia faces acute shortages of skilled professionals and has a low physician-to-population ratio of 2.5 physicians per 100,000. The World Health Organization (WHO) recommendation for low-income countries is four times higher at 10 physicians per 100,000 [6–9]. The insufficiency of the workforce to meet the demand for care results in the inability to provide sufficient and proper care [10, 11]. Such human resource challenges are further exacerbated by policy and regulatory incoherence and inconsistencies in Ethiopia and, more generally, SSA [6, 12].

Ethiopian public hospitals especially reflect the country’s poor health system and deal with a persistent human resources crisis, even by SSA standards [7]. The government has taken notable steps to reform the health system, especially in the domain of maternal and child health [10, 13]. The reforms have, however, fallen short of resolving the contextual challenges hospitals encounter in the human resource management (HRM) domain. First, the policy measures have been unable to resolve the labour market issues arising from an insufficient supply of skilled healthcare professionals and difficulties with retaining the qualified staff attracted [4, 14, 15]. Second, public hospital reform is hampered by a heritage of poor general and administrative performance [16–18]. Third, institutional health system issues hamper effective reform implementation, despite the robust regulatory framework put in place [16, 17]. Among these institutional challenges are budget constraints and centralized decision making, which are subject to political dynamics [8, 13, 14, 19].

Our research aims to understand how such contextual challenges influence HRM in public hospitals that deliver health services to the Ethiopian population. More specifically and formally, the research question regarding Ethiopian public hospitals therefore is as follows: How do contextual health system mechanisms influence hospital-level HRM in Ethiopian public hospitals?

Before presenting the theoretical framework and methods to address this research question, we present further background on Ethiopia, its health system and its public hospitals.

## Background

### Ethiopia and the Ethiopian health care system

The population of Ethiopia was approximately 120 million by the end of 2021, of which 83% lived in rural areas [20]. Human resource shortages and imbalances, large geographical distances, budget shortages, and socioeconomic factors are the main causes of substandard and poor health services for the Ethiopian population [21]. Even according to SSA standards, Ethiopia experiences relatively high burdens of disease and mortality. Respiratory infections, maternal and child health complications, road traffic injuries, malaria, tuberculosis, and diarrhoeal diseases are among the conditions with the highest disease burden and years of life lost [22–24].

**Fig. 1:**
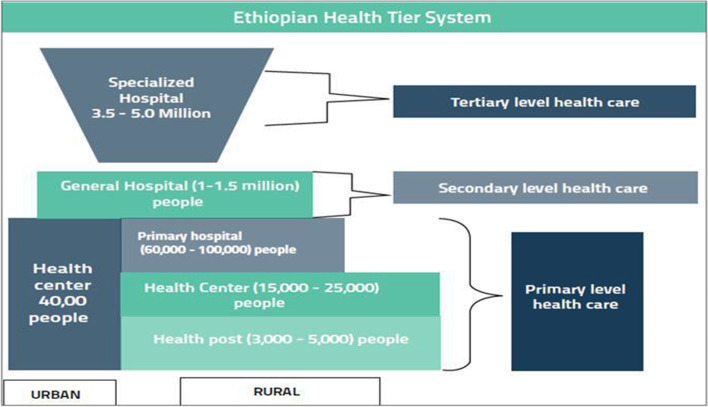
The Ethiopian Health Care System. Source: (Federal Ministry of Health, 2019 [17]; Alebachew & Waddington, 2015 [26])

The Ethiopian health system is comprised of primary-, secondary-, and tertiary-level facilities. The primary level encompasses primary hospitals, health centres, and health posts. Secondary-level (general) hospitals and tertiary-level (university) hospitals manage complex heath conditions for larger populations [25]. The resulting three-tiered system is depicted in Fig. 1, which also depicts the hospital catchment population sizes per level [13].

The total Ethiopian health workforce amounted to 219,542 in 2016, of which 150,534 (68%) were health professionals of various categories for the entire health system [13]. The Federal Ministry of Health (MOH) plans to progressively increase the number of health professionals in various categories from 248,538 in 2020 to 374,368 by 2025, which is still well below the WHO recommendation of 2.3 health workers per 1000 population set for SSA [10].

Reforms to increase these numbers have been negatively impacted by job dissatisfaction at all levels of the Ethiopian public health system, causing the workforce to leave for jobs in private healthcare, other sectors, and/or abroad. Low salary, poor working conditions, and poor compensation and benefit packages are the main factors pushing’ the workforce to seek employment outside of the Ethiopian public health system [4, 27]. The reforms not only have struggled to effectively address job satisfaction and staff motivation but have more generally failed to improve skill levels and the quality and efficiency of service delivery [9]. Hence, there is an urgent call for developing an effective HRM strategy and fostering a healthy, committed, and respectful workforce to address skill gaps, motivation, satisfaction, migration, and incentives and thereby improve patient outcomes [7]. Correspondingly, Ethiopia’s ‘strategic human resources for health plan’ (2016–2020) sets a vision for an adequate number of well qualified, committed, compassionate, respectful, and caring health workers contributing to the public health sector vision of Ethiopia and policy goals [13].

### Literature and theoretical framework

HRM can be defined as a systematic approach to manage the workforce aiming to optimize performance of employees [28]. Effective HRM has been reported to be of critical importance in healthcare organizations (e.g., [29, 30]). A recent systematic literature review identified evidence on the effectiveness of HRM practices in SSA hospitals [2]. Although HRM varies in different settings, effective HRM practices can increase client satisfaction and, more generally, enhance organizational performance through their effect on employee behaviour [28, 31]. Strategic HRM (SHRM) aligns HRM and business strategy in which HRM is designed to enable an organization to achieve its goals and enhance firm performance and competitive advantage is achieved [32, 33].

Several SHRM models have been developed over the years, each with its own focus and strengths, for example, the Huselid model, Guest model, Brewster model, Michigan model, Harvard model, Sparrow model, and Warwick model [34]. Several classic SHRM models focus on the elements between HRM and outcomes (e.g., Guest model, Michigan model). Most models have broadened the scope by including several (internal and external) contextual factors that influence SHRM (e.g., the Harvard model). However, the extent and detail to which contextual factors are emphasized varies among models. Some models combine different models and/or theories into one framework. For example, in the Contextual SHRM Framework, Paauwe combines several theoretical perspectives into one framework. It shows that HRM is part of an organization, which is part of a broader society/operating context. The Contextual SHRM Framework focuses on three broad contextual mechanisms that affect the SHRM system adapted by an organization: competitive, institutional, and heritage mechanisms. The Contextual SHRM Framework, which is the improved version of the contextually based human resource theory (CBHRT) framework (both developed by Paauwe) [35–38], has been adopted by a variety of authors to understand the effects of context on strategic HRM issues, both within the healthcare sector and in other sectors [38–41].

To uncover the role contextual challenges play in the strategic HRM of Ethiopian public hospitals, the authors build on the Contextual SHRM Framework (by Paauwe) and on recent insights into SSA hospitals [21]. In addition, the study is mindful of the limitations to the validity of the Contextual SHRM Framework in the context of public hospitals in Ethiopia. For instance, a topic such as competitive advantage may be (ir) relevant in ways that are not yet considered. Moreover, this context may reveal factors not yet included in the framework.

## Methods

**Fig. 2:**
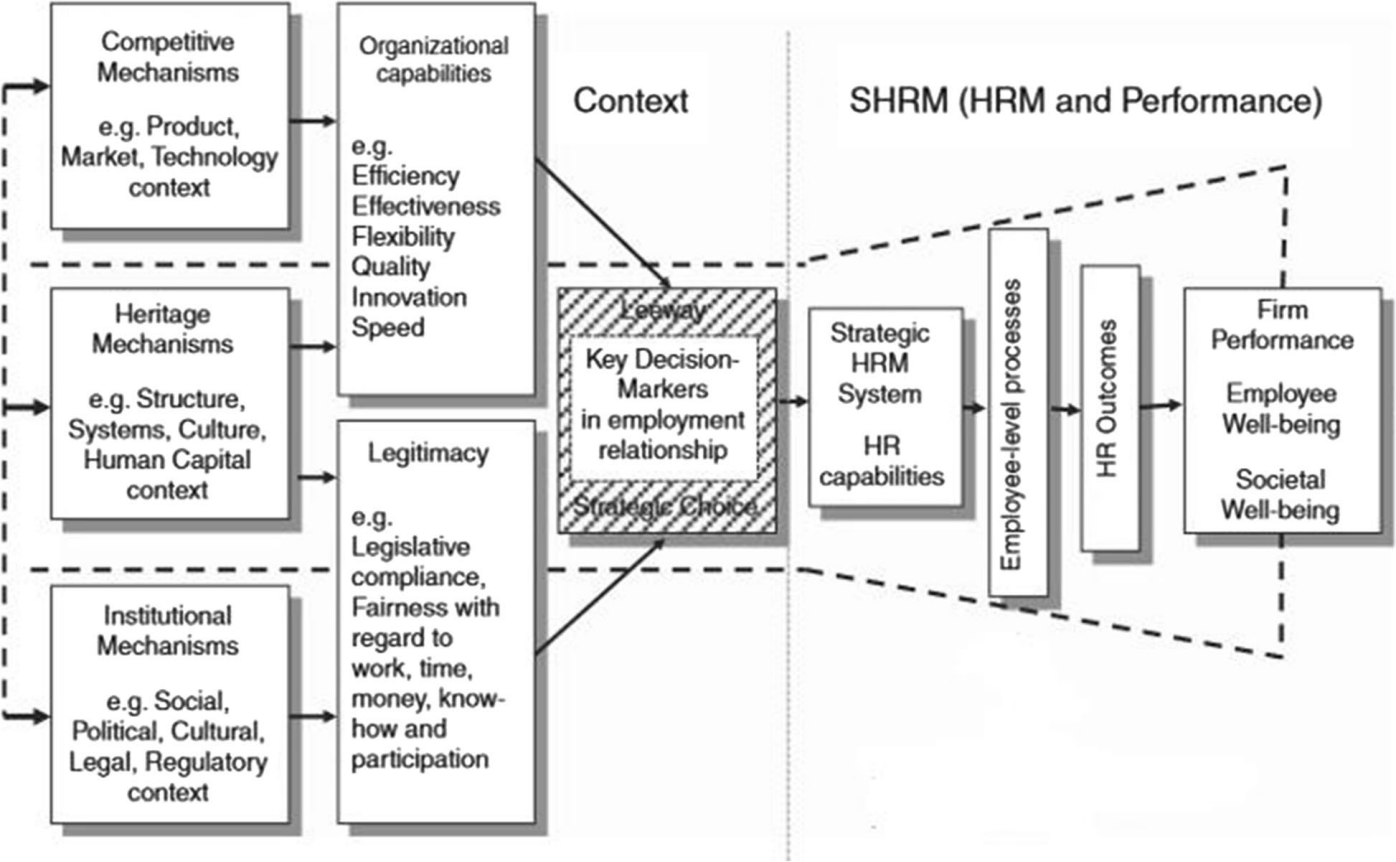
Contextual SHRM Framework (source: Paauwe and Farndale 2017 [37])

A qualitative study was conducted through structured interviews with respondents from public hospitals in Ethiopia. The study findings are reported in accordance with the COREQ criteria (see Additional file 2 )[42]. The interview structure was derived from Paauwe’s Contextual SHRM Framework [37] (see Fig. 2).

### Data collection

The authors selected 15 hospitals by purposive sampling. We aimed to select public hospitals that are representative of the Ethiopian public setting. Authors thus approached a collection of hospitals that differed in hospital level (i.e., general, teaching, specialized, primary), geographical setting (i.e., regional large towns, regional and rural provincial settings, city government of Addis Ababa), and governance (i.e., federal and regional level governments). Table 1 gives details of all the hospitals included for data collection. Data were collected between March and September 2019.

**Table 1:** Hospital characteristics selected for data collection

Hospital name	Hospital Level	Geographic Location	Governing body (Public)	Established	Beds
Dupti	General	Afar Region	Regional Gov’t/Health Bureau	1958	124
Arbaminch	General	SNNP Region	Regional Gov’t/Health Bureau	1961	238
Wolaita	General teaching	SNNP Region	Central Gov’t: Federal MOH&MoSHE	1928	347
Alert	General	Addis Ababa City	Central Gov’t: Federal MOH	1934	300
St Peter	Specialized	Addis Ababa City	Central Gov’t: Federal MOH	1953	300
Ghandi	Specialized	Addis Ababa City	City Government	1958	144
Ras Desta	General	Addis Ababa City	City Government	1931	200
St Paul’s	Specialized teaching	Addis Ababa City	Central Gov’t: Federal MOH & MoSHE	1969	700
Adama	General teaching	(East) Oromia Region	Regional Gov’t/Health Bureau	1946	400
Mojo	Primary	(East) Oromia Region	Regional Gov’t/Health Bureau	2015	80
Ambo	General	(West) Oromia Region	Regional Gov’t/Health Bureau	1946	88
Guder	Primary	(West) Oromia Region	Regional Gov’t/Health Bureau	2015	50
Hawassa	Comprehensive specialized teaching	SNNP Region	Central Gov’t: Federal MOH&MoSHE	1998	350
Tulla	Primary	SNNP Region	Regional Gov’t/Health Bureau	2017	80
Shashemene	Specialized	(South) Oromia Region	Regional Gov’t/Health Bureau	1948	306

*NB* MoSHE refers to Ministry of Science and Higher Education, *MOH* stands for Ministry of Health, *Gov’t* stands for Government, *SNNP* stands for Southern Nations, Nationalities and People

Interviews were conducted with purposively selected respondents holding different positions within the hospital’s HRM hierarchy who are knowledgeable of the hospital’s HRM practices. This enabled us to gain broad insight into the variety of viewpoints on the contextual mechanisms that influence HRM across departments and hierarchical levels.

Our first request at each hospital was to interview the executive board member responsible for HRM and the head of the HRM department (Table 2 shows the respondents’ characteristics). If these respondents were not able to participate or if these positions were not fulfilled within the structure, team leaders and HR directors of health bureaus were approached. In total, the authors interviewed 19 respondents.

**Table 2:** Respondents characteristics interview

**Region**	Addis Ababa	6
	Afar Region	2
	Oromia Region	5
	SNNP Region	6
**Hospital Type**	Specialized	5
	General	11
	Primary	3
**Position**		
	CEO	6
	Head department	13
**Educational background**	Bachelor degree	9
	Master degree	8
	Medical doctor	2
**Current organizational tenure**	< 5 years	3
	5–10 years	6
	10–20 years	5
	20–30 years	4
	> 30 years	1
**Years in current position**	< 5 years	17
	5–10 years	1
	10–20 years	1
	20–30 years	0
	> 30 years	0
**Total respondents**		**19**

In addition, the authors conducted 4 focus group discussions (FGDs) with 38 participants in total. The participants of the FGDs (details presented in Table 3) were managers, experienced middle managers and line managers, including matrons, heads of the clinical, outpatient, and inpatient departments, and heads of departments responsible for quality, governance, and planning. The FGDs provided a second source of data from middle and lower management respondents, ensuring data triangulation.

**Table 3:** Focus Group Discussion participants characteristics

**Region**	Addis Ababa	10
	Afar Region	10
	Oromia Region	6
	SNNP Region	12
**Total**	4	38

The topic list and structured interview guideline (presented in Appendix 1) were jointly constructed by all authors and based on document analysis of the FMOH Health Account and National HRH strategic Plan [13, 17] and Paauwe’s framework [37]. The interview guideline was piloted in three Ethiopian hospitals by the first and second authors and subsequently revised. To ensure quality and validity, all authors were involved in this stage. The second author (JVK) travelled three times to visit the first author (PPG), where the research was conducted, while the third author engaged from the Netherlands (MBS).

### Data analysis

All interviews and FGDs were audio-recorded and transcribed verbatim after ensuring written consent from all respondents. The transcripts were thematically analysed using ATLAS.ti 8 [43]. We followed our original, approved, protocol which provided several forms of data triangulation. The analysis followed the steps below, in which all authors were involved:

- Step 1 The authors familiarized themselves with the data by (re) reading transcripts and identifying the essence and patterns of meaning and potential issues of interest.
- Step 2 An initial coding tree or scheme was developed to generate topics of interest. These initial codes were identified following a deductive coding approach based on the Conceptual SHRM Framework.
- Step 3 The authors verified whether the initial list of codes covered the key elements of Paauwe’s model and resolved any gaps. Moreover, the authors inductively generated open codes emerging from the data that did not appear to directly relate to the contextual SHRM framework (such as poverty and moonlighting).
- Step 4 Broader code groups were created for each theme, and subgroups of codes were created for code groups with a large number of codes.
- Step 5 All codes were combined into agreed-upon broader code groups and themes that were based on similarities and (visualized) linkages in the data and the framework.
- Step 6 The final themes were analysed and synthesized into results, presented below.

Ethical approval was obtained from the Ethiopian Public Health Institute.

## Results

This section presents the main findings of our analysis in a sequence that chronologically provides evidence to answer the research questions described in the introduction. The results are structured to show how the three contextual factors, competitive, institutional and heritage mechanisms, are linked to and positively or negatively influence HRM and health outcomes in Ethiopian public hospitals.

### Competitive mechanisms: competing for resources and not for patients

As defined in Paauwe’s framework, this mechanism entails the product (e.g., service provisioning), market (competitiveness and economic fitness) and technology (innovation) contexts.

None of the respondents mentioned competition for patients. Neither were hospitals perceived to lose patients for reasons of quality or (limitations in) services provided: *‘We don’t lose customers because there is no competition (R8).’* This is likely caused by insufficient capacity to fully meet the demand for care, as can be witnessed in the treatment of admitted patients. For example, *‘Emergency departments have patients on recliners for several days* (R11).’ As a result, hospitals appear to compete for scarce resources rather than for customers. Respondents particularly mention the competition for skilled health care professionals. Rural hospitals are most challenged in this competition: *‘Rural hospitals face a critical shortages of nurses, surgeons, radiologists, laboratory technicians, and ENT staff (R17).’* Likewise, public hospitals are perceived to struggle more than private hospitals, which offer better working conditions, such as a higher salary.

Innovation was hardly recognized as a relevant competitive factor. Most respondents reported little to no innovation due to budget shortages, high patient demands and government influence. Among the few exceptions mentioned in health service innovation are the introduction of renal transplantation and the placement of shipping containers to resolve room shortages (R5).

Interestingly, some hospitals have taken innovative approaches to improve their attractiveness as employers, again focusing on the competition for resources. For instance, they offered benefits for their employees, such as free medical services for all staff, a supermarket for personnel, or transportation services. In addition, hospitals tried to enhance their attractiveness as employers through HRM innovations such as increasing autonomy and job security, providing education, and introducing collaborative leadership (R11).

### Institutional mechanisms: the dominant role of the government

The institutional mechanisms mentioned in Paauwe’s framework are 1) institutional isomorphisms that influence decision making in organizations, 2) coercive mechanisms that emerge from power sources (e.g., government, employment legislation), and 3) normative mechanisms of adopting standards. They include sociocultural values and norms and the policy, legal and political context impacting strategic HRM practices. The findings for this component relate almost exclusively to the government.

The studied hospitals are subject to coercive pressures that result from government regulations. For instance, respondents frequently mention the influence of stringent regulations. Government regulations are perceived as set in stone and ‘*are executed like the Quran and the Bible’* and pervasively impact daily practices in ‘*hiring, salary, allowances, promotion, firing, disciplinary measures, as HR managers have no leeway to change them [regulations].’* In exceptional cases, these stringent and pervasive regulations are seen as beneficial and supportive. For instance, regulations to increase maternity leave from 3 to 4 months were perceived as positive, as they improved the quality of life of female workers (R16).

In general, however, respondents considered governmental regulation to be counterproductive. This is especially the case for regulations regarding financial incentives where payment differences that resulted from regulations were perceived as unfair. The respondents considered it to be especially discriminatory if payment differences occurred in cases perceived as comparable:

‘*Regulations are not supportive; there is variation in implementing the regulations and law on workforce deployment, salary and allowances even in the same region (R12).’*

FG2 members considered that members of the *‘workforce with the same level of occupation, profession and experiences are compensated with different salary levels that violate labour rights, which is discouraging for HR managers and workforce.’*

Respondents also explicitly mentioned differences in financial regulation between medical doctors, nurses, and other hospital staff as being unfair, discriminatory, and disproportional. FG3, for example, added*: ‘professionals’ work is enabled by the support from a nonclinical cohort of staff as a team, but the regulation violates such teamwork through unfair allowances.’*

Some respondents pointed out that *‘ … such differences negatively impact collaboration and team building in hospitals, reduce well-being, and might cause turnover of skilled professionals* (R 12).’ ‘*Teamwork is coerced rather than built organically via these discriminatory practices* (R10).’ FG2 participants shared this view: ‘*Although* h*ospital care is teamwork, government regulation violates this culture through discouraging compensation*.’

All the studied hospitals reported tension between political versus hospital interests and goals. Politicians are perceived to set up goals for hospitals that mainly refer to productivity in numbers, including numbers of health service professionals and support staff. The activities by which the government subsequently allocates increasing numbers of professionals and staff is sometimes referred to as an HRH flooding strategy. For instance, respondents mentioned the ‘*flooding strategy for physicians*’ (R3) and the ‘*massive production of nurses’ as enforced by politicians ….*’ (R10). Respondents perceived that this quantitative focus inhibited attention to the quality of healthcare services for patients and for the well-being of the workforce. Hence, in the eyes of many respondents, politicians prioritized policy interests and political interests, such as party interests, over hospital interests.

*‘*…. *[There is] political imposition to focus on quantity over quality. This has induced gaps in the knowledge and skills of professionals (R19’).*

*‘Their [politicians’] conflicting interest of fighting for their political issues and maintaining their top position and loyalty to party interest. No attention given for managing hospital and service quality (R3).’*

In some cases in which politicians held executive positions in the hospital, political interests were reported to be better aligned with the hospital interests. Compared to their competitors, these hospitals appeared to benefit from political involvement:

*‘Our primary hospital is lucky because it has the local-level politicians as board members, and party loyalty made them empowered and confident. They [the politicians] are very supportive with the budget, but at the federal level and in some regions, there is no such political support in budget and HRM issues (R17).’*

Normative mechanisms emanating from government regulations affect HRM practices. All the studied hospitals report that government regulation does not accept or allow absenteeism and moonlighting. However, some respondents considered that *‘although moonlighting is an unacceptable norm [to the government), it is practised mostly by skilled professionals because of coercive mechanisms of the government (e.g., not allowing workforce to get equal overtime payment and allowances) (R16).’ ‘This pressures professionals to illegally engage in dual practices or causes high turnover (R1).’* The resultant high turnover might subsequently put the quality of care at risk:

*‘Hospitals are a human capital-intensive and risky working environment, but a risk allowance incentive set by policy makers/government for the whole workforce of the hospital was not considered [by management] (FG3).’*

*‘This is because the regulations failed to address the low salary, which is pushing professionals to leave and negatively impacts service provision and health outcomes (R1).’*

Despite the dissatisfaction with the current policy and the felt urgency to change it, some respondents had faith in the improvement attempts. In general, however, respondents stated that the policy reforms aimed at managing budgets and the allocation of the (newly educated) workforce and disregarded the well-being and job satisfaction of the workforce in the public hospitals.

Variation in the implementation of regulations between regions further decreased faith in policy and further increased the dissatisfaction of the workforce:

*‘There are variations in implementing health policy, with a lack of health insurance for employees in some regions. It is present in other regions and hospitals, with a clear policy and HR strategy incoherence and inconsistency in the country. This leads to apathy, low satisfaction and performance of the workforce (FG3).’*

### Heritage mechanisms: A heritage of limited human resource management leeway.

Paauwe’s framework describes heritage mechanisms affecting the human capital context, organizational culture, structure, and systems that ultimately impact HRM. It considers the path dependence of HRM and its fit with other preceding organizational developments.

From the responses, it becomes clear that the aforementioned lack of competition and top-down enforcement of stringent regulations are long standing and have therefore become part of HRM practices. Even on the operational level, the studied hospitals report that staffing issues have been controlled not by hospital management but by ministries and/or regional health bureaus. For example,

*‘The role of the HR manager is not recognized because the structure doesn’t empower the HR department, mainly due to failures of health policy reform in addressing HR issues (FG2).’*

All hospitals reported a prolonged lack of leeway to develop their own HRM systems and practices. Respondents perceived the regulatory bureaucracy as a complex contextual and organizational structure that hinders the resolution of HRM challenges and promotes political dynamics.

*‘The structure of teaching hospitals is very complex and confusing, with accountability to various government bodies and multisector governance from federal ministries (R5).’*

‘*The governing board is not supportive in addressing hospital demands and HR issues in time; instead, an intersectoral governance approach is missing (FG3).’*

Within this difficult and complex environment, leadership was found to be a critical element. However, it was felt that leaders mostly adopted the government logic and were disconnected from HRM and patient care:

*‘Our hospital lacks leadership competence, which also contributes to the inability to improve HR issues. This is mainly due to the appointment procedure based on party criteria (FG2).’*

Some respondents reported a *‘Lack of a supportive leadership culture in valuing staff as an asset also creating disengagement of workforce (R12).’ ‘Ex-leadership was autocratic, giving more focus to ethnic/tribal and political networks, unable to solve HR problems (R6).’*

Positive affirmations of the importance of leadership were also provided: *‘Our hospital has a culture of collaborative leadership in empowering line managers to take HR responsibilities (R11).*’ *‘The hospital values the workforce as an asset rather than a cost (R15). ‘There is a culture of collegial relationships that are useful for employee and hospital performance; there is a new culture developed by the CEO of the hospital with a good staff-management relationship (R1).’*

## Discussion

To understand how empirically identified contextual factors influence HRM practices in Ethiopian public hospitals, we conducted qualitative research based on the Contextual SHRM Framework. This framework includes the institutional mechanisms (e.g., policy, legal, and regulatory frameworks and sociocultural, demographic, and political factors), competitive mechanisms (e.g., labour market, technology, and innovation), and heritage mechanisms (e.g., structure, culture, systems, and human capital), as identified to impact SHRM in Ethiopian public hospitals. In addition to this deductive approach, we inductively searched for additional contextual factors within these three mechanism categories and explored the working of these factors. The results are based on document analysis, individual interviews, and focus group discussions.

Before addressing competitive, heritage, and institutional mechanisms separately, a main first finding is that the persistent shortages of human resources and financial resources form a foundational contextual factor that influences most competitive, institutional, and heritage mechanisms. The national government actively engages to address the consequences of these shortages by allocating human and financial resources to specific hospitals. Moreover, it promotes the education of increased volumes of health professionals. This government policy reform and implementation subsequently forms a main institutional factor that severely impacts HRM in Ethiopian hospitals, as further addressed below.

With respect to competitive mechanisms, the scarcity of human resources leads to supply-side competition for skilled professionals. This competition is particularly challenging for public hospitals, which depend on governmental allocation decisions and rarely have leeway to deviate from the prescribed salaries and allowances to retain necessary skilled personnel. Hence, jobs in private hospitals and outside the health sector continue to attract public hospital staff, causing turnover at public hospitals to be high. This limits the effectiveness of the HR flooding and allocation strategies implemented by the government, while hospitals have little room to manoeuvre to resolve these problems. These findings confirm previous research reporting a shortage of skilled professionals aggravated by high turnover and mainly driven by budget scarcity and corresponding low salaries [6, 7]. Previous studies (e.g., [9, 10]) have also shown that the current government efforts may not lead to a decrease in workforce shortages unless financial resources are addressed. Moreover, the practice of allowing moonlighting might continue to form an attractive and common, yet noncompliant, human resource practice. This situation contrasts with that in other African countries, in which moonlighting is regulated and accepted [44].

Our findings thus provide evidence of human resource shortages faced by low- and middle-income countries, causing the market to be eminently shaped by service supply [15]. Our data do not provide evidence of competitive mechanisms on the demand side, e.g., hospitals competing for patients by providing higher quality of care or additional services. Correspondingly, the few innovative practices we found mainly aimed to increase the attractiveness of the hospital for employees and were not targeted at patients. They included offering free medical and transportation services for employees and HRM innovations (e.g., increasing job autonomy, job security, education) and introducing collaborative leadership.

The dominant institutional factor of tight government control was perceived to be quantity oriented. This finding substantiates previous research [7] on government responses to workforce shortages. Moreover, the tightly controlling regulations influenced HRM at a very detailed level, such as the number of nurses per department and the salaries of individuals. This tight quantitative control implied logics and priorities that differed considerably from the views of the hospital employees. These professionals prioritized quality over quantity, in particular the quality of care provided and the quality of the arrangements for human resources. Such differences in logics between management and professionals have been previously recognized to be negatively associated with retention and thus to adversely impact the underlying policy aims [4].

From a human resource perspective, and in view of the low salaries, differences in regulation and arrangements for allowances and external employments were a main concern. They caused financial inequalities among employees that were perceived as unjust and led to dissatisfaction and demotivation. Some studies [16, 19] support our finding that differences in regulations, political forces and shortages of critical resources complicate attempts to address urgent HRM issues. Previous studies added financial inequalities, lack of coordination and ineffective policy implementation as causes for incoherence and regulatory/policy failures to address HRM issues [45–47].

The focus on quantity diminished the possibility of tailoring HRM arrangements to the needs of employees. Therefore, this approach contrasts sharply with the HRM architecture and talent management literature, which stresses the need for tailoring HRM practices for organizational performance [48]. The combination of a dominant top-down government logic and regulation that was ‘implemented like the Koran and the Bible’ with the difficulties experienced by staff as communicated bottom up put the hospital and HR leadership in a difficult position. HR managers felt compelled to devote their time to the implementation of and compliance with tight regulations. These regulations inhibited the empowerment of hospital and HR leaders to provide tailored and locally effective responses as considered necessary to effectively address the challenges. Leaders experienced a lack of autonomy. In terms of the contextual SHRM framework, they perceived a lack of ‘leeway’ or ‘room to manoeuvre’ [35].

The lack of leeway and the corresponding HR leadership challenges were perceived as a long-lasting heritage factor. There is a history of government appointment of senior hospital management. Board members were often perceived to emerge from a network with political, tribal, and/or religious ties and to prioritize the logic and demands from this background over workforce logic and demands. While it was understood that the needs and objectives from the government and the workforce were very difficult to address simultaneously, their perceived inability to address workforce demands contributed to senior management/leadership being regarded as incompetent. Likewise, the literature shows that managers’ lack of decision space/autonomy for changing HRM also contributes to the perceived low competence of leaders and the hospital workforce [49–52]. In addition. The workforce appeared to have rarely been engaged in HRM decision making. The lack of bottom-up approaches has led to apathy, disengagement, and demotivation and subsequently to illegal moonlighting and high turnover.

The results of our study clearly emphasize the relevance of some of the categories and elements of the contextual SHRM framework, such as government-driven coercive and normative institutional factors. Competitive mechanisms played a role, yet only on the financial and human resource supply side, rather than on the demand side of health service competition. These factors differ from the empirical findings underpinning the contextual SHRM framework. Likewise, our findings reveal few of the traditional heritage mechanisms. They do reveal a history of leadership challenges, however, which relate partially to appointment practices and partially to the effects of the combined contextual mechanisms: a lack of leeway in HRM. The very complex HRM challenges faced by Ethiopian hospitals are addressed in a strategic HRM context where management has very little room to manoeuvre. This might explain why practices have emerged that are technically outside this regulated space, such as moonlighting, in an effort to retain the skilled staff needed to provide public health services to the Ethiopian population. These findings reveal HRM practices that are beyond the leeway captured by the Contextual SHRM Framework [35].

### Strengths and limitations of the study

This study includes a large and varied sample of Ethiopian hospitals covering various geographic locations, rural and urban settings, and central and regional governments. The study particularly engages various respondents, ranging from experienced administrators and HR managers to team leaders and professionals. This triangulation was further strengthened by using interviews, focus group discussion, and document analysis.

After piloting and contextualization, the structured interviews following the Contextual SHRM Framework elicited rich responses from the respondents and captured the external mechanisms influencing HR management in Ethiopian hospitals. Moreover, the complementary open questions and inductive analysis enabled us to identify additional factors and insights. Our study therefore adds to previous studies that were largely based on secondary data, gave little attention to these external factors and addressed other contexts (e.g.,[2, 3, 19]).

A first limitation of our study concerns the regional conflicts within Ethiopia that caused delays and restricted travel to the study settings/regions (Oromia, SNNP). Moreover, the recent circumstances in the large, diverse and dynamic federal state Ethiopia have impacted the data collection opportunities and caused us to adhere to our approved research protocol. We cannot claim to have covered all current perspectives nor to have reached data saturation. We may have missed some factors and aspects of mechanisms, and our study may not be generalizable to other hospitals in Ethiopia. Second, the collection of data from employees not involved in management was not extensive (only as FGD participants). More extensive inclusion of such respondents is recommended in future research (see, e.g., [4]). Third, quantitative data for some HRM and management issues (e.g., workforce satisfaction and perception towards engagement) were not accurately documented in each of the hospitals. Thus, data triangulation through the addition of quantitative data was not possible. Fourth, the study exclusively focused on public hospitals in Ethiopia. Therefore, the generalizability to private hospitals and other healthcare contexts and countries may be low.

## Conclusions

Guided by Paauwe’s Contextual SHRM framework, our study looked at how competitive, institutional and heritage mechanisms influence the shaping of SHRM in fifteen public hospitals in Ethiopia. It is considered that the combination of these contextual factors shapes SHRM in the studied hospitals. The competitive mechanisms relate mostly to competition for scarce human resources rather than for customers and rarely to competitive advantage and innovation. The institutional mechanisms appear most important and influence SHRM through stringent top-down regulations, supporting governmental policies to build workforce volume within a limited budget. The heritage mechanisms reveal little variation between public hospitals and appear to be mainly entrenched with politics and government regulations regarding health workforce policies.

Our study shows that although top management complied with the coercive government regulations/policies, these instruments failed to address persistent HRM challenges. Hospitals lack autonomy to design their HRM policy/strategy and tailor arrangements to workforce needs. Leadership is perceived to lack competence, as HR managers lack leeway to shape HRM.

As potential remedies for the aforementioned situations, we therefore recommend the following:

- The government should loosen regulations and provide authority and leeway to hospitals for strategic HRM to tailor solutions to the local context and challenges.
- The governmental entities involved should collaboratively design simplified organizational and HR governance structures, especially for teaching hospitals.
- Government regulations can more actively consider health service demand and promote responsiveness in provisioning service delivery and service quality, thus connecting explicitly to the values of the professionals and the needs of patients.

## Supplementary Information

**Additional file 1. **Supplementary materials (Appendix 1) are available as Additional File 1.

**Additional file 2. **Consolidated criteria for reporting qualitative research (COREQ): a 32-item checklist for interviews and focus groups.

## Data Availability

The datasets used and/or analysed during the current study available from the corresponding author on reasonable request.
